# Optical properties of drug metabolites in latent fingermarks

**DOI:** 10.1038/srep20336

**Published:** 2016-02-03

**Authors:** Yao Shen, Qing Ai

**Affiliations:** 1 School of Forensic Science, People’s Public Security University of China, Beijing 100038, China; 2 Department of Physics, Applied Optics Beijing Area Major Laboratory, Beijing Normal University, Beijing 100875, China

## Abstract

Drug metabolites usually have structures of split-ring resonators (SRRs), which might lead to negative permittivity and permeability in electromagnetic field. As a result, in the UV-vis region, the latent fingermarks images of drug addicts and non drug users are inverse. The optical properties of latent fingermarks are quite different between drug addicts and non-drug users. This is a technic superiority for crime scene investigation to distinguish them. In this paper, we calculate the permittivity and permeability of drug metabolites using tight-binding model. The latent fingermarks of smokers and non-smokers are given as an example.

In 1968, negative index material (NIM) was first introduced by Veselago^1^. NIM can have negative permittivity and permeability simultaneously. Pendry *et al*.^2^,^3^,^4^,^5^,^6^ gave a deep discussion and pointed out that a configuration which was called split-ring resonator (SRR)^3^ could put negative refraction into practice, apart from some particular configurations with non-trivial symmetry breaking^7^. From then on, negative refraction became a focus in scientific research, for example NIM can be used to fabricate perfect lenses to enhance local field and detection sensitivity^8^,^9^,^10^,^11^. Two years later, Shelby *et al*.^12^ realized NIM experimentally. Metamaterial, made up of SRRs or molecules which consist of SRRs, e.g. extended metal atom chains, becomes a new branch of study^13^,^14^,^15^,^16^,^17^,^18^,^19^,^20^,^21^,^22^,^23^,^24^,^25^,^26^,^27^,^28^,^29^,^30^,^31^,^32^,^33^,^34^,^35^,^36^,^37^. Many new directions are developed, such as electromagnetic cloaking^38^,^39^,^40^,^41^,^42^,^43^, toroidal moment^44^, liquid crystal magnetic control^45^, etc. On the other hand, in forensic science, on highly reflective surface, the latent fingermarks are difficult to be observed. The traditional method of visualizing the invisible fingermarks is using fluorescent tag. In Boddis and Russell’s paper^46^, they made use of antibody-magnetic particle conjugates to visualize them. During this procedure, they find the latent fingermarks of smokers and non-smokers are quite different. As a result, the latent fingermarks of these two kinds of donors are observed to be inverse and thus they can be used to identify the smokers. The research of distinguishing drug users by metabolites becomes a new focus in forensic science field^47^,^48^. In this paper, we first introduce negative refraction phenomenon to forensic science. We point out that when we put those latent fingermarks of drug addicts and non-drug users in the light field, they can also be identified. Furthermore, our method is physical and non-damaged, because the latent fingermarks will not be destroyed. More importantly, due to quantum effect, a small volume of molecules could sufficiently respond negatively to the applied electromagnetic fields^49^. We give the theoretical derivation and calculation of this phenomenon. Our result is not only suitable for smokers but also for drug addicts. In other words, except for cotinine, the metabolite of nicotine, benzoylecgonine and morphine can also be detected using our method.

## Results

### Tight-binding Approximation and Hückel Model

Many molecules of drug metabolites have a broken ring configuration, i.e. SRR. This structure gives them special optical properties. Without loss of generality, we calculate cotinine, i.e. the metabolite of nicotine, as an example.

**Figure 1:**
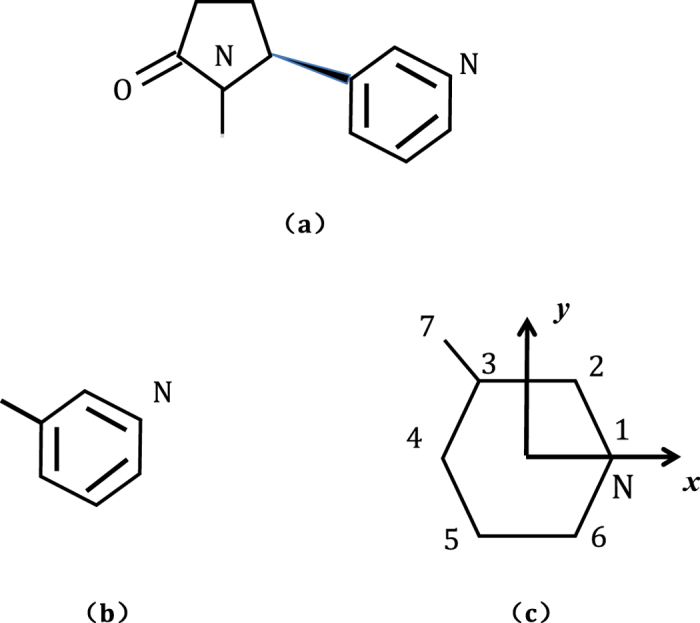
(**a**) The chemical structure of cotinine molecule (*C*_10_*H*_12_*N*_2_*O*). Cotinine has two rings, one is pyridyl, and the other is pyrrolidine. (**b**) The simplified model. The molecule is simplified into a pyridyl and a methyl. (**c**) The spatial distribution of atoms in the simplified model. The origin is set at the center of the hexagon. Seven sites are labeled sequentially. Site 1 is a nitrogen atom and others are carbon atoms.

Figure 1(a) demonstrates the structure of cotinine molecule. The main part of cotinine is the hexagon part which is called pyridine as shown in Fig. 1(b). In this part, one carbon atom of the ring is substituted with one nitrogen atom. For the sake of simplicity, the remaining part of cotinine molecule is simplified as a methyl in the same plane. This simplification is reasonable because the main contribution to the optical property comes from the *π* electrons in the conjugate part of cotinine molecule, i.e. single-nitrogen-substituted heterocyclic annulene^50^ or 3-methylpyridine in Fig. 1(c). Hückel calculation is justifiable and sufficient for the 3-methylpyridine model, though it is a simplified model. Recently, by using a small set of empirical parameters, the Hückel method has been successfully applied to calculate the energy of highest-occupied *π* orbital and the first *π* − π^*^ transition energy for a large set of organic molecules with less than 13% deviation. As a consequence, we utilize the Hückel model with the set of empirical parameters to simulate the optical properties of cotinine molecules in our paper. Alternatively, we also notice that by taking *σ* orbital into account, extended Hückel theory^51^,^52^,^53^,^54^ may provide better result with much less consuming time as compared to other more accurate method, e.g. time-dependent density function theory.

*π* electrons of the pyridyl interact with the electromagnetic fields and therefore result in special optical properties of cotinine. The structure of 3-methylpyridine consists of one pyridyl and one methyl with sites labeled as in Fig. 1(c). Although there are seven electrons in 3-methylpyridine, it has been discovered that one of the electrons is mainly located at the methyl and does not contribute to the current in the ring. Because the magnetic response originates from the circular current, hereafter we shall restrict our calculation to the pyridyl. However, due to the presence of methyl, two carbon-carbon bond lengths have been slightly modified. The problem starts from toluene (methylbenzene) which adds a methyl on a benzene. The quantum dynamics of six *π* electrons of benzene are described by the Hückel model as^55^


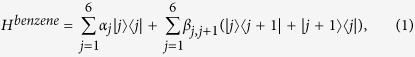


where *j* is the site label, 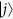 denotes the state with a *π* electron at site *j, α*_*j*_ is the site energy and *α*_*C*_ = −6.7 *eV*^56^. The coupling strength between *j*th and (*j* + 1)th sites is given by the resonant integral 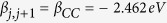. Here we use Harrison expression^57^,^58^


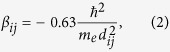


where 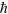 is the Planck constant, *m*_*e*_ is the mass of electron, *d*_*ij*_ is the bond length and can be attained from NIST^56^,^59^. Benzene has four energy levels *ε*_1_, *ε*_2_, *ε*_3_, *ε*_4_, which are labeled sequentially from the lowest eigen energy. Both the degeneracies of *ε*_2_ and *ε*_3_ are two, while *ε*_1_ and *ε*_4_ are non-degenerate.

For toluene, the resonant integrals of benzene have been modified, because the bond lengths are revised by the presence of the methyl. The bond length of benzene is *d* = 1.397 *A*, while toluene have two values, i.e. *d*_1_ = 1.394 *A* and *d*_2_ = 1.395 *A*^56^,^59^. The smaller one is the bond length between carbon which is connected to the methyl and its adjacent atoms. The revised resonant integrals are *β*_1_ = −2.469 *eV* and *β*_2_ = −2.473 *eV*. As labeled in Fig. 1(c), the Hückel Hamiltonian of toluene reads


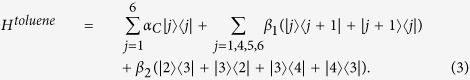


**Figure 2:**
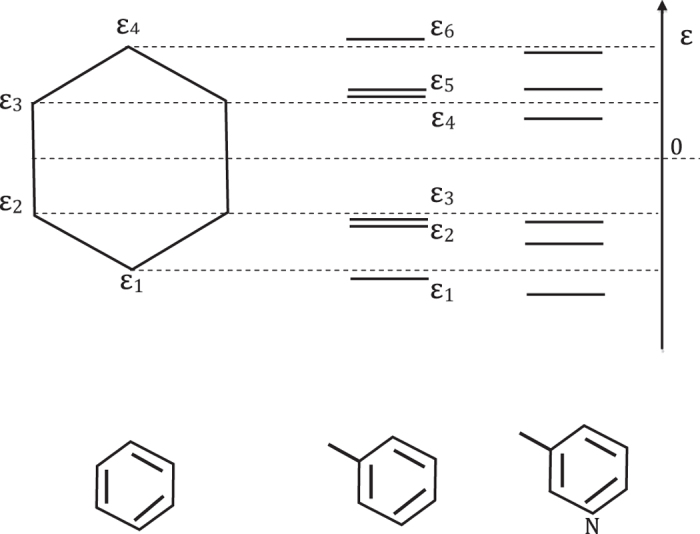
The energy spectra of (left) benzene, (middle) toluene and (right) simplified cotinine molecule.

In this case, the energy spectrum of toluene can be solved exactly as


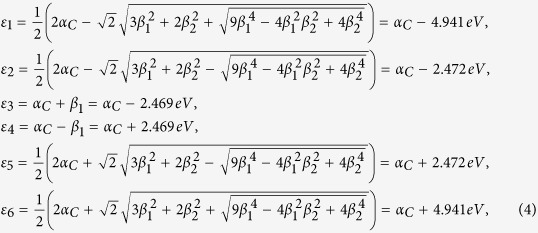


which are schematically shown in Fig. 2. And we do not list the eigen vectors here for simplicity. Because of the methyl, the degeneracy is lifted.

For simplified cotinine, the Hamiltonian is described as


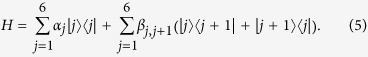


The nitrogen atom is located at site 1, cf. Fig. 1(c). In this configuration, the site energies and coupling constants are explicitly given as


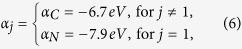



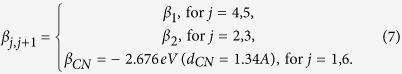


By diagonalization, the Hückel Hamiltonian (5) can be reexpressed as


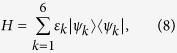


where 
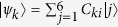
 is *k*th single-electron molecular orbital, *ε*_*k*_ is the eigen energy.

In order to obtain 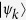 and *ε*_*k*_, we use the perturbation theory in quantum mechanics. We assume that the unperturbed system is a toluene. Then, the perturbation originates from


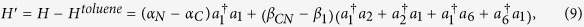


where 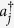 is the creation operator on *j*th site and *a*_*j*_ is the annihilation operator. As a consequence, the simplified cotinine molecule has also six energy levels (see Fig. 2), and all of them are non-degenerate. Hereafter, for the sake of simplicity, we further assume *α*_*C*_ = 0.

According to the perturbation theory, the energy spectrum of the simplified cotinine molecule reads


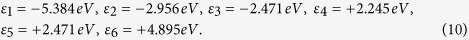


Following the degenerate perturbation theory, we can obtain the wave function to the first order. Since their explicit expressions are very complicated, we do not list the analytical expressions of energy and wave function here.

**Figure 3:**
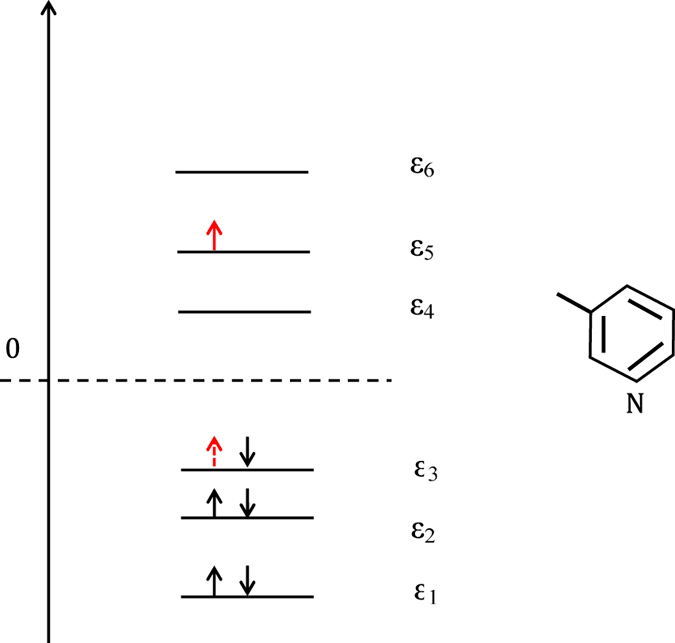
Schematic for the single-electron excitation of cotinine molecule (red solid up-arrow) from the ground state (red dashed up-arrow), i.e. 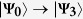 with transition energy Δ*E*_3_ = *E*_3_ − *E*_0_ = *ε*_5_ − *ε*_3_.

The cotinine molecule has six non-interacting *π*-electrons. On account of the spin degree (see Fig. 3), the ground state can be expressed in the second-quantization form as


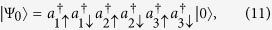


and *E*_0_ represents the ground-state energy of the whole cotinine system, i.e.


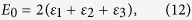


where 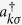 is the creation operator of the orbital *k* with spin *σ* 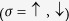.

The system has eighteen single-excitation states, for example, the 2th and 3th excited states are


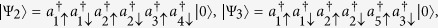


with corresponding eigen energies


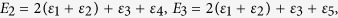


respectively. In the first case, the electron with energy *ε*_3_ and spin down is excited to energy level *ε*_4_ with spin conserved. In the second case, the electron with energy *ε*_3_ and spin up is excited to energy level *ε*_5_ with spin conserved. Here the flip of electronic spin is not taken into consideration. To sum up, the single-excitation states read


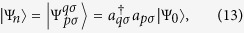


where *p* = 1, 2, 3, *q* = 4, 5, 6, 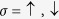, and the eigen energies are


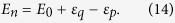


In the subspaces spanned by the ground state and single-excitation states, the Hamiltonian without electro-magnetic field reads


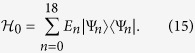


### Perturbation Theory in Electromagnetic Field

When there is a time-dependent electromagnetic field applied on the molecule, based on the dipole approximation, the total Hamiltonian including the interaction between the molecule and the electromagnetic field can be written as


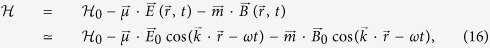


where 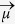 and 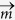 denote the electric and magnetic dipole moments respectively. By assuming the spatial scale of the molecule is much smaller than the wave length of the field 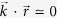 since the coordinate is chosen as Fig. 1(c), we have


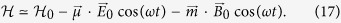


By a unitary transformation


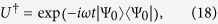


the Hamiltonian becomes time-independent


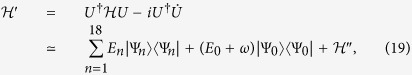


where


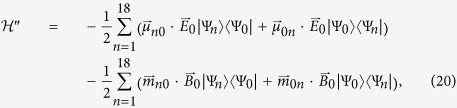



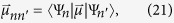



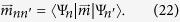


In other words, we change the system from the static frame into a rotating frame. In the rotating frame, the state and operator become 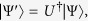 and 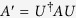, respectively. Moreover, due to the interaction with electromagnetic field, the molecular ground state becomes


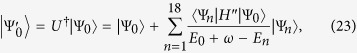


#### Permittivity

The electric dipole moment in the rotating frame reads


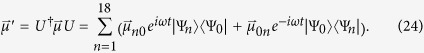


For the ground state, the expectation value for the dipole operator in the rotating frame is


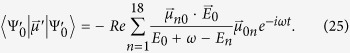


In the electromagnetic field, the electric displacement field in a volume *V* with *N* identical molecules


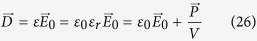


reads


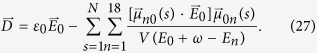


Thus, the total permittivity in different direction is


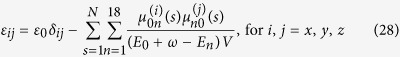


The relative dielectric constant of the system, i.e. the permittivity, gives


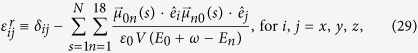


where 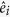 is the unit vector of the lab coordinate system.

Because we choose the symmetric center of pyridyl as the origin of coordinate, the electric dipole moment reads


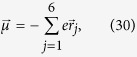


where 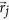 is the vector of *j*th electron and −*e* is the electric charge of an electron. Because 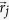
’s are single-electron operators, the matrix elements of electric dipole operators are given by


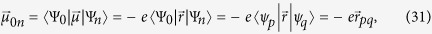


where 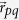 is the overlap of 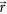 between two single-electron wave functions, i.e.


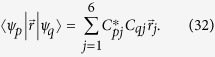


#### Permeability

To account for the magnetic response of cotinine molecule, we start from the Heisenberg equations of motion,


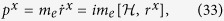



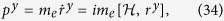


where we assume *ħ* = 1.

The magnetic dipole moment is related to the angular momentum of the system. The angular momentum operators read


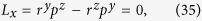



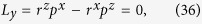



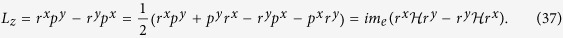


Obviously, only the response in *z* direction is present as all atoms in the cotinine molecule are restricted in the *xy* plane. Therefore, the magnetic dipole moment is


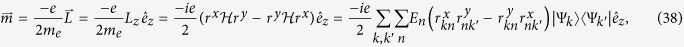


where


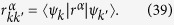


Similar to the electric response, the expectation value for the magnetic dipole operator in the rotating frame is


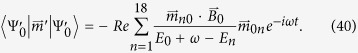


The magnetic induction in a volume *V* with *N* identical molecules


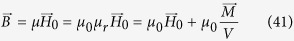


is explicitly given by


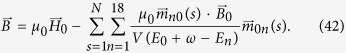


Notice that *μ* is the permeability of medium, different from the electric dipole moment 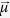 above.

The relative permeability of cotinine medium is simplified as


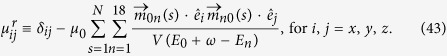


#### Analysis

Equations (29) and (43) present the analytical results for the relative permittivity and permeability of cotinine molecules in electromagnetic field. According to the expressions of these two quantities, they can be negative simultaneously when the second parts of the expressions are greater than unity. In order to fulfill this requirement, the denominators of the second parts should be small enough. In other words, 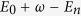 needs to be much smaller than numerator which means 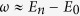. For a given initial energy of the electron before transition *E*_0_, we can observe simultaneous negative permittivity and permeability of cotinine molecules in electromagnetic field when the driving frequency *ω* is tuned approximately equal to the transition frequency 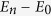.

### Numerical Simulation of Permittivity and Permeability

**Figure 4:**
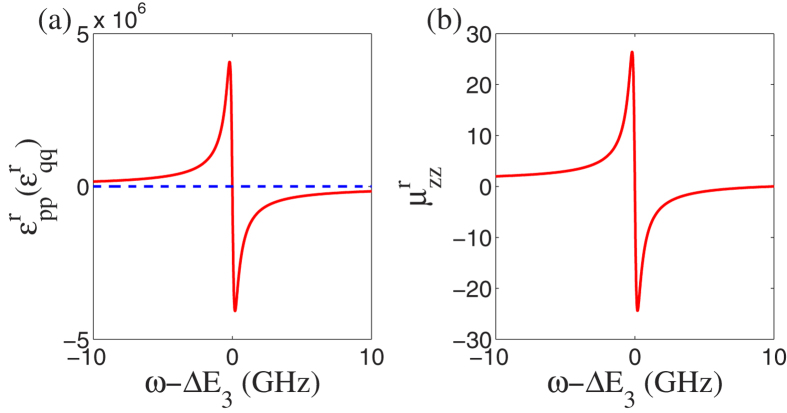
The numerical results of (a) permittivity and (**b**) permeability of cotinine molecules vs the light frequency *ω*. The permittivity 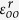 (red solid line) along one main axis in the *xy* plane is negative near the resonance frequency, while 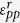 (blue dashed line) along the other main axis is always constant. In the magnetic response, the permeability along *z* direction 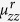 is shown.

In the above section, the analytical derivation suggests that relative permittivity and permeability of cotinine molecules might be negative simultaneously in certain frequency regime. Here we show and analyze the numerical result. In the investigated model, the cotinine molecule is simplified as a pyridine and a methyl, c.f. Fig. 1(b). The simplified cotinine model is of two dimension. Thus, we only need to analyze the electromagnetic responses of the molecules in two directions. Figure 4 shows the numerical simulation of relative dielectric constants in the *xy* plane and relative magnetic permittivity in *z* direction of the system. Here we assume the site energies *α*_*C*_ = 0, *α*_*N*_ = *α*_*C*_ − 1.2 *eV*, and the coupling strengths *β*_*CC*_ = −2.462 eV, revised coupling strengths *β*_1_ = −2.469 eV, *β*_2_ = −2.473 eV, *β*_*CN*_ = −2.676 *eV*^55^,^57^,^58^,^59^. The excited-state life time *τ* = 10 ns is within the range of experimentally observation, e.g. 90 *μ*s^60^. For a transition to the first excited state, e.g. a spin-up electron is excited from *ε*_3_ to *ε*_4_, the contributions from transition dipoles *μ*_01_ and *m*_01_ are much larger than others i.e., 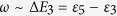. In Fig. 4(a), both relative dielectric constants in the two main axes 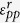 and 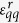 are different from unity in the vacuum case, as the presence of nitrogen atom breaks the reflection symmetry along the axis connecting site 3 and the origin. Furthermore, Fig. 4 clearly shows the negative permittivity and permeability at the same time. This result suggests that cotinine molecules can be detected by negative refraction.

## Discussion

In this paper we research the optical properties of drug metabolites in latent fingermarks. All of these drug metabolites have a structure in common, i.e. SRR which could realize negative refraction. And negative refraction makes the optical properties of latent fingermark quite different between drug addicts and non-drug users and thus can be used to distinguish them. Illuminated by the same incident field, the latent fingermarks of these two kinds of donors may be observed in the different directions with respect to the normal of the interface. The method is to print the donor’s fingermarks on the transparent media and to observe them in the light transmission direction on the opposite side with respect to the side for the normal refraction. In the ordinary case, the refracted light and incident light are on the opposite sides of the normal. However, if the donor is a drug addict, we can detect the refracted light on the same side of incident light with respect to the normal. Although the concentration of drug metabolites may not be evenly distributed in the fingermark, some parts of the fingermark can be detected by negative refraction once the concentrations of drug metabolites in these parts are sufficiently large. Because of negative refraction, the fingermarks of drug addicts can be distinguished from those of non-drug users. Without loss of generality, we take cotinine as an example to calculate electromagnetic response of metabolites in latent fingermarks of smokers. According to our analytic derivation and numerical simulation, we demonstrate the presence of negative refraction in cotinine molecules. The advantage of this method is that it is physical and non-damaged. Our method is suitable for all drug metabolites which have the SRR structure. And this method can also be conveniently applied to distinguish drug addicts and non-drug users. For example, except for cotinine, benzoylecgonine and morphine can also be detected using our method.

## Additional Information

**How to cite this article**: Shen, Y. and Ai, Q. Optical properties of drug metabolites in latent fingermarks. *Sci. Rep*. **6**, 20336; doi: 10.1038/srep20336 (2016).
